# Commentary: Sugar Metabolism Regulates Flavor Preferences and Portal Glucose Sensing

**DOI:** 10.3389/fnint.2019.00004

**Published:** 2019-02-14

**Authors:** Anthony Sclafani, Karen Ackroff

**Affiliations:** Department of Psychology, Brooklyn College of the City University of New York, Brooklyn, NY, United States

**Keywords:** flavor preferences, glucose sensing, intestines, portal vein, vagus, dopamine

In an interesting paper by Zhang et al. (2018), flavor conditioning effects of intragastric (IG) infusions of glucose and a non-metabolizable glucose analog (α-methyl-D-glucopyranoside, MDG) were compared in mice. Infusions of both sugars during one-bottle training stimulated intake of their associated flavored non-nutritive drinks compared to control mice infused with water. This confirmed Zukerman et al. (2013a), who reported that glucose and MDG not only stimulated intake but also conditioned flavor preferences relative to a water-paired flavor. In contrast, IG infusion of fructose did not stimulate intake or condition a flavor preference. Glucose and MDG, unlike fructose, are ligands for intestinal sodium glucose co-transporters/sensors (SGLTl, SGLT3), which implicates these sensors in flavor conditioning. Pharmacological blockade of SGLTs prevented MDG conditioning, whereas blockade of both SGLTs and GLUT2 was required to prevent glucose conditioning. In another study, genetic deletion of SGLT1 blocked MDG and glucose flavor preference conditioning (Sclafani et al., 2016).

Zhang et al. (2018) reported that mice preferred a glucose-paired flavor over an MDG-paired flavor in a direct choice test. The same result was observed in duodenal bypass mice in which the IG sugar infusions emptied into the jejunum, which the authors took as evidence for a post-absorptive glucose action. In support of this view, hepatic-portal vein infusions of glucose, but not MDG, increased extracellular dopamine in ventral and dorsal striatum. They concluded that portal sensing of glucose metabolism via the hepatoportal-brain neural axis is the “preferential physiological pathway for sugar reward” and excluded a role for circulating “gut factors.” These conclusions, however, are not fully supported by findings of other investigators.

In rats, duodenal and jejunal glucose infusions conditioned similar flavor preference whereas ileal infusions were ineffective; yet infusions at all three sites increased blood glucose levels (Ackroff et al., 2010). Furthermore, portal glucose infusions in rats did not condition preferences for flavored saccharin solutions, nor did intraperitoneal glucose infusions in mice (Ackroff et al., 2010; Zukerman et al., 2013b). Thus, elevation in circulating glucose, by itself, is not an adequate stimulus for flavor conditioning. However, portal glucose infusions conditioned a preference for flavored chow (Tordoff and Friedman, 1986), which suggests that portal glucose is an effective conditioning stimulus when combined with pre-absorptive nutrient stimulation. Consistent with this interpretation, portal glucose infusions conditioned preferences for flavored glucose but not for flavored saccharin solutions (Gowans, 1992; Ackroff et al., 2010). These results do not support the primacy of portal glucose sensing in post-oral sugar reinforcement but suggest instead that portal sensing enhances the reinforcement actions of intestinal glucose sensing. Ren et al. (2010) reported that IG glucose infusions increased striatal dopamine release. Thus, the enhanced flavor conditioning produced by glucose may result because the pre- and post-absorptive actions of the sugar promote a greater dopamine response than does the pre-absorptive action of MDG. The conditioning actions of the non-metabolizable MDG may also be limited by its accumulation in intestinal cells: unlike glucose, MDG is not actively transported out of the cells by GLUT2. Consistent with this possibility, as the concentration of IG infused sugar increased, MDG-conditioned preferences decreased whereas glucose-conditioned preferences increased (Zukerman et al., 2013b).

Zhang et al. (2018) assumed that portal glucose metabolism generated a signal that triggered striatal dopamine release. This may be the case, but it should be noted that the hepatic-portal region contains glucose sensors, including SGLT3, which can detect glucose independent of its metabolism (Mithieux, 2014). Portal MDG infusions did not stimulate dopamine release, which excludes SGLT3 in this response. The GLUT2 glucose transporter is also implicated in portal glucose sensing which may be secondary to increased glucose metabolism, but a direct role for GLUT2 as a glucose sensor cannot be excluded (Thorens, 2015). Thus, it remains to be determined whether portal glucose stimulation of striatal dopamine is secondary to glucose metabolism.

How reinforcing signals, generated by peripheral glucose and MDG, reach the brain is uncertain. Zhang et al. (2018) dismissed gut circulating factors and implicated a hepatoportal-brain neural pathway. But they failed to consider reports that disrupting neural afferents by abdominal vagotomy, selective afferent vagotomy, or capsaicin treatment did not block glucose-conditioned flavor preferences (Lucas and Sclafani, 1996; Sclafani and Lucas, 1996; Sclafani et al., 2003; Zukerman et al., 2011). In addition, vagotomy does not prevent the rapid activation by IG glucose infusions of brain reward sites implicated in flavor preference learning (Tsurugizawa et al., 2009). Portal sensors signal the brain via sympathetic fibers (Bohland et al., 2014); disrupting these fibers attenuates but does not block glucose-conditioned preferences (Sclafani et al., 2003). Thus, it is premature to rule out a role for gut humoral factors in glucose- and MDG-conditioned flavor preferences.

In summary, the Zhang et al. (2018) study provides new information on the role of portal glucose sensing in the enhanced potency of glucose to condition flavor preferences relative to MDG. However, the suggestion that portal glucose sensing is more significant than intestinal sensing in post-oral sugar reinforcement effects is not consistent with the available literature.

## Author Contributions

All authors listed have made a substantial, direct and intellectual contribution to the work, and approved it for publication.

### Conflict of Interest Statement

The authors declare that the research was conducted in the absence of any commercial or financial relationships that could be construed as a potential conflict of interest.
